# Concentration and time-dependent amyloidogenic characteristics of intrinsically disordered N-terminal region of *Saccharomyces cerevisiae* Stm1

**DOI:** 10.3389/fmicb.2023.1206945

**Published:** 2023-10-19

**Authors:** Venkata Subbaiah S P, Patil Pranita Uttamrao, Uttam Das, Sruthi Sundaresan, Thenmalarchelvi Rathinavelan

**Affiliations:** Department of Biotechnology, Indian Institute of Technology Hyderabad, Kandi, Telangana, India

**Keywords:** *Saccharomyces cerevisiae*, Stm1, intrinsically disordered protein, amyloid fibril, apoptosis-like cell death, triplex binding protein, quadruplex binding protein

## Abstract

*Saccharomyces cerevisiae* Stm1 protein is a ribosomal association factor, which plays an important role in preserving ribosomes in a nutrition-deprived environment. It is also shown to take part in apoptosis-like cell death. Stm1 N-terminal region (Stm1_N^1-113^) is shown to recognize purine motif DNA triplex and G-quadruplex. Circular dichroism (CD) spectra of Stm1_N^1-113^ (enriched in positively-charged Lysine and Arginine; negatively-charged Aspartate; polar-uncharged Threonine, Asparagine, Proline and Serine; hydrophobic Alanine, Valine, and Glycine) collected after 0 and 24 h indicate that the protein assumes beta-sheet conformation at the higher concentrations in contrast to intrinsically disordered conformation seen for its monomeric form found in the crystal structure. Thioflavin-T kinetics experiments indicate that the lag phase is influenced by the salt concentration. Atomic force microscopy (AFM) images collected for a variety of Stm1_N^1-113^ concentrations (in the range of 1–400 μM) in the presence of 150 mM NaCl at 0, 24, and 48 h indicate a threshold concentration requirement to observe the time-dependent amyloid formation. This is prominent seen at the physiological salt concentration of 150 mM NaCl with the fibrillation observed for 400 μM concentration at 48 h, whereas oligomerization or proto-fibrillation is seen for the other concentrations. Such concentration-dependent fibrillation of Stm1_N^1-113^ explains that amyloid fibrils formed during the overexpression of Stm1_N^1-113^ may act as a molecular device to trigger apoptosis-like cell death.

## Introduction

Stm1 is a 273 amino acids long protein located in the 12th chromosome of *Saccharomyces cerevisiae*. Stm1 participates in several vital cellular events. It is shown to play a role in apoptosis-like cell death (Ligr et al., 2001), preservation of ribosomes under nutrient deprivation environment (Van Dyke et al., 2006, 2013), telomere maintenance (Hayashi and Murakami, 2002; Van Dyke et al., 2004), and promoting mRNA decapping and degradation (Balagopal and Parker, 2009). Stm1 protein is abundant in the cytosol in association with free 80S ribosome, and a smaller fraction is also present in the nucleus (Van Dyke et al., 2004).

Stm1 is a well-known purine motif DNA triplex (Nelson et al., 2000) and quadruplex binding protein (Frantz and Gilbert, 1995; Van Dyke et al., 2004). Specifically, the N-terminal region of Stm1 (Stm1_N^1-113^) is shown to interact with the purine motif triplex (Katayama et al., 2007). Further, Stm1_N^1-113^ is found to have a role in the preservation of ribosomes under a nutrition-deprived environment (Ben-Shem et al., 2011; Van Dyke et al., 2013). Indeed, the Stm1 N-terminal region occupies the mRNA binding tunnel of the ribosome (PDB ID: 4 V88), thus providing the molecular basis for its role in translation inhibition during the nutrient deprivation environment (Ben-Shem et al., 2011). In the ribosome-bound monomeric form, the Stm1 N-terminal region is in the intrinsically disordered state, along with three short alpha-helical fragments (Ben-Shem et al., 2011; Fernandez et al., 2013; Garreau de Loubresse et al., 2014; Mailliot et al., 2016; Prokhorova et al., 2016, 2017; Melnikov et al., 2016a,b; Pellegrino et al., 2019).

Elucidating the secondary structural information of Stm1_N^1-113^ apo-form may provide a clue(s) about the molecular basis of its diverse biological functions. Thus, the secondary structural characterization of Stm1_N^1-113^ is carried out here using circular dichroism (CD) experiments. Surprisingly, the intrinsically disordered Stm1_N^1-113^ shows a beta-sheet characteristics in a concentration-dependent manner. Thioflavin-T (Th-T) emission spectra, Th-T kinetics, and atomic force microscopy (AFM) experiments confirm the amyloid fibrillation of Stm1_N^1-113^. AFM images further show that Stm1_N^1-113^ fibrillation occurs via spherical and rod-shaped protofibril intermediates. Thus, the results presented here would be helpful in understanding the biological role of the amyloidogenic characteristics of Stm1_N^1-113^.

## Results

### Stm1_N^1-113^ takes up concentration-dependent conformations: intrinsically disordered to beta-sheet conformation

**Figure 1:**
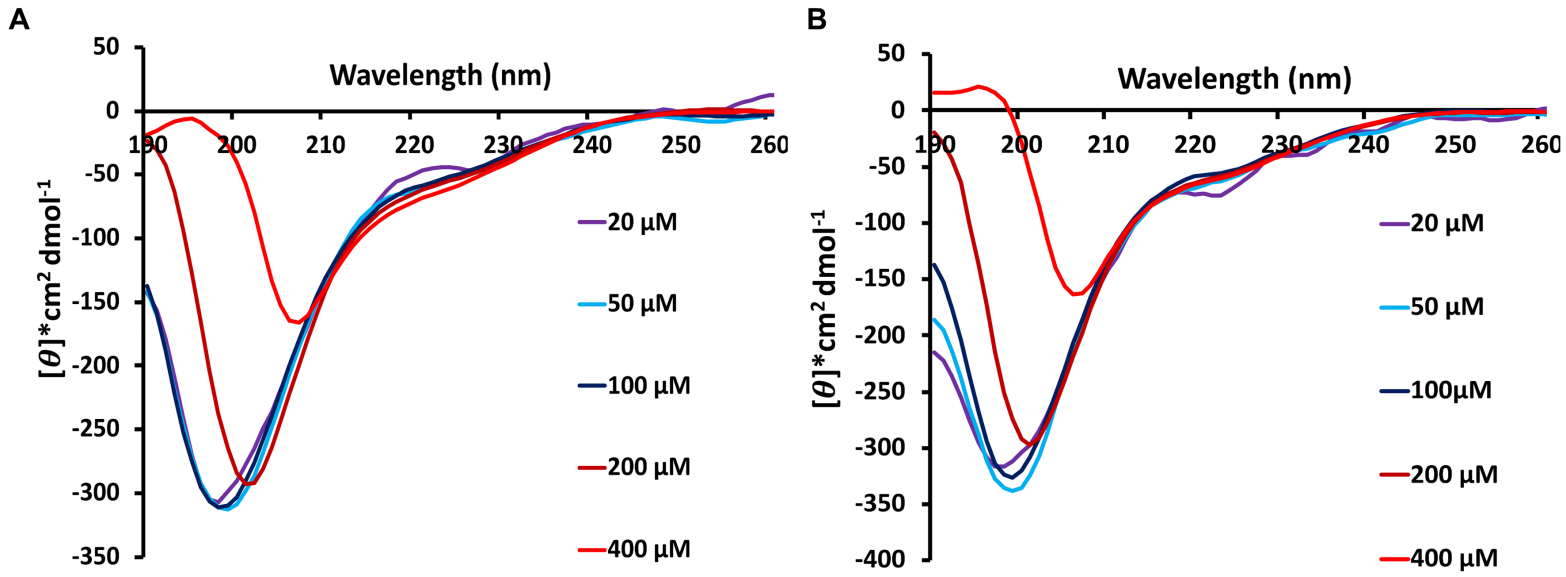
Circular dichroism spectra showing the concentration and time-dependent amyloid forming characteristics of Stm1_N^1-113^ in the presence 10 mM NaCl at **(A)** 0 h and **(B)** 24 h. 20, 50, 100, 200, and 400 μM concentrations of Stm1_N^1-113^ are used for the data collection (spectra collected using 0.1 mm pathlength cuvette). Note that the spectra are color-coded based on the different concentrations of Stm1_N^1-113^. See text for details.

Circular dichroism spectra collected at 0 h reveal that Stm1_N^1-113^ takes up an intrinsically disordered conformation at the lower concentration of 20 μM in the presence of 10 mM NaCl (Figure 1A). The negative peak in the 195–200 nm wavelength region is seen at 20 μM concentration (Figure 1). Such a negative peak falls at ~200 nm for 100 μM Stm1_N^1-113^ concentration (Figure 1A). However, at 200 μM concentration, the negative peak falls between 200 and 205 nm. The negative peak further moves to 208 nm at 400 μM concentration. Along with this, a positive peak starts appearing at 195 nm at 400 μM concentration (Figure 1A). CD spectra collected at 24 h reveal that the β-sheet conformation is pronounced at 24 h, specifically at the 400 μM concentration of Stm1_N^1-113^, as the positive peak emerges around 195 nm (Figure 1B). These indicate that Stm1_N^1-113^ undergoes a transition from random coil to β-sheet formation in the presence of 10 mM NaCl, which is influenced by its concentration.

### Th-T emission and Th-T kinetics spectra reveal amyloid formation at the higher concentrations of Stm1_N^1-113^

**Figure 2:**
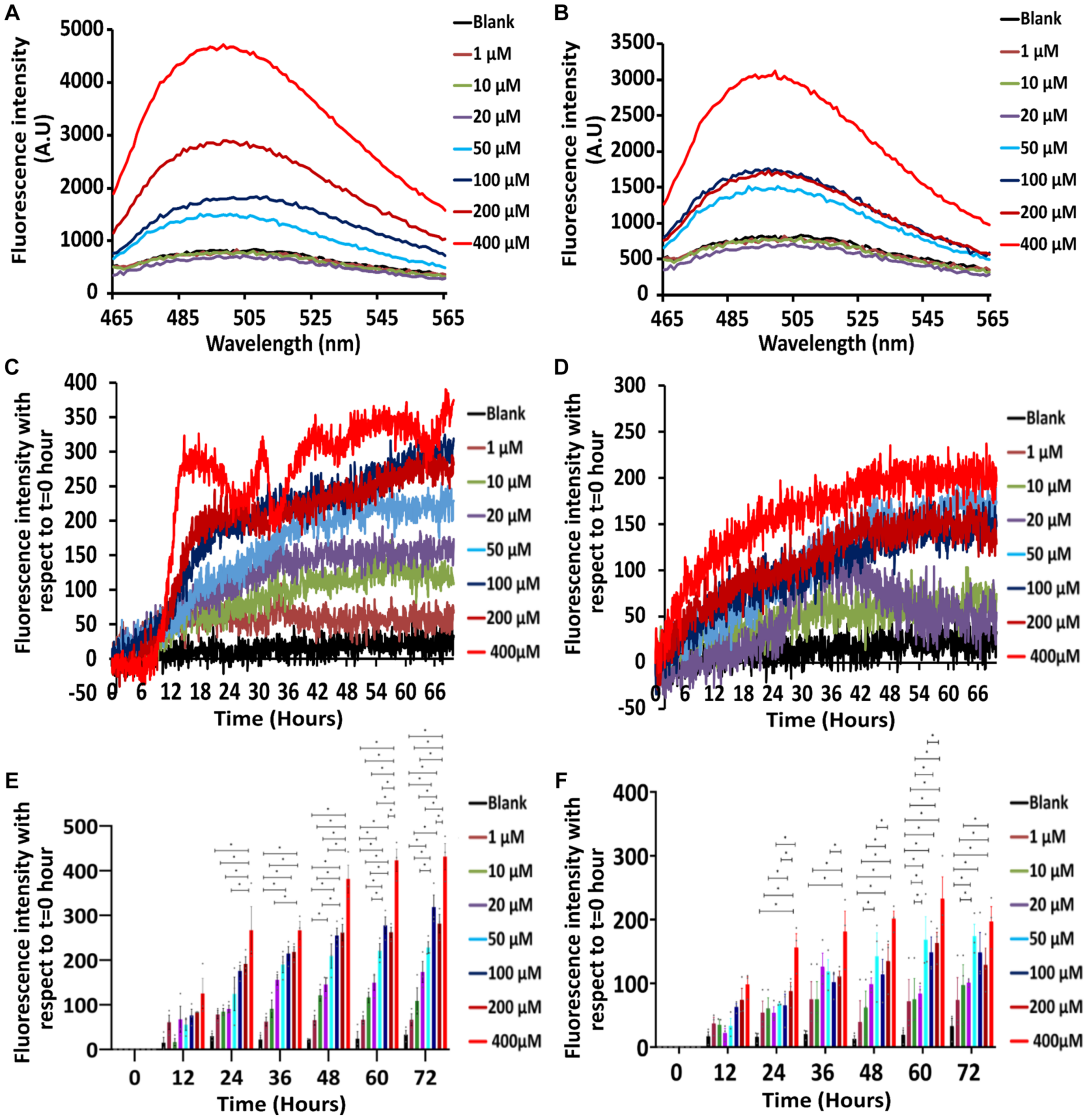
Th-T emission and kinetics experiments showing the concentration and time-dependence of Stm1_N^1-113^ amyloid formation at two different salt concentrations. **(A,B)** Th-T emission spectra corresponding to the titration of 20 μM Th-T with 1, 10, 20, 50, 100, 200, and 400 μM concentrations of Stm1_N^1-113^ in the presence of 10 mM NaCl **(A)** and 150 mM NaCl **(B)**. Note the minimum concentration requirement of 50 μM to exhibit the amyloidogenic characteristics. See text for details. The wavelength (nm) is represented in the X-axis, and the fluorescence intensity [arbitrary unit (A.U)] is represented in the Y-axis. The spectra are color-coded based on the different concentrations of Stm1_N^1-113^. **(C,D)** Th-T kinetics data (collected for 72 h) corresponding to different concentrations of Stm1_N^1-113^ (1, 10, 20, 50, 100, 200, and 400 μM) at the abovementioned two different NaCl concentrations [10 mM **(C)** and 150 mM **(D)**] after the titration with 20 μM Th-T. See text for details. The time (h) is represented in the X-axis and the fluorescence intensity is represented in the Y-axis. The spectra are color-coded based on the different concentrations of Stm1_N^1-113^. **(E,F)** Fluorescence intensity bar diagram illustrating the concentration effect on Stm1_N^1-113^ aggregation in the presence of 10 mM **(E)** and 150 mM NaCl **(F)** at different protein concentrations (1, 10, 20, 50, 100, 200, and 400 μM) in a time interval of 12 h during 0–72 h. Statistical significance analyzed with one-way ANOVA using the Tukey *post hoc* test is indicated (*p* ≤ 0.05) along with standard error of the mean (SEM). Note that the corresponding data points (sample size = 3) are indicated in filled circles.

Since CD experiments (Figure 1) have shown the tendency of the protein to take beta-sheet conformation, the ability of the protein to form amyloid conformation is explored using Th-T assay in the presence of 10 mM (Figures 2A,C,E; Supplementary Figure S1A) and 150 mM NaCl (Figures 2B,D,F; Supplementary Figure S1B) for different concentrations of Stm1_N^1-113^.

The Th-T emission spectra indicate that there is no prominent peak seen around 495 nm for Th-T blank as well as for the lower concentrations of Stm1_N^1-113^ in the presence of 10 mM NaCl (1, 10, and 20 μM; Figure 2A). However, from 50 μM Stm1_N^1-113^ concentration onwards, there is an increase in the fluorescence intensity at ~495 nm. Further, there is an increase in the fluorescence intensities (~495 nm) with respect to increasing concentrations of Stm1_N^1-113^ (50, 100, 200, and 400 μM). This is an indication of amyloid fibril formation at 50, 100, 200, and 400 μM Stm1_N^1-113^ concentrations. Strikingly, the spectra corresponding to 400 μM Stm1_N^1-113^ have the highest fluorescence intensity, followed by 200 μM Stm1_N^1-113^ concentration. In line with this, CD spectra also shows a shift in the negative peak toward the higher wavelength (*viz.,* above 200 nm), specifically at the higher concentrations (Figure 1).

Th-T kinetics experiments carried out till 72 h further confirm the amyloidogenic tendency of Stm1_N^1-113^ at 50, 100, 200, and 400 μM concentrations in the presence of 10 mM NaCl concentration (Figure 2C). While 1 μM Stm1_N^1-113^ emission spectra overlap with the blank, the spectra corresponding to 10 and 20 μM Stm1_N^1-113^ concentrations show a slight increase in the fluorescence intensity, and the spectrum corresponding to 50 μM Stm1_N^1-113^ concentration show a further increase in the fluorescence intensity. The fluorescence intensity of spectra corresponding to 100 and 200 μM Stm1_N^1-113^ exhibit a nearly similar fluorescence intensity. The Th-T kinetics assay also shows the highest fluorescence intensity at 400 μM Stm1_N^1-113^ concentration, consistent with the Th-T emission spectra. There is a steep increase in the fluorescence intensity till ~15 h, beyond which it attains a saturation at 400 μM Stm1_N^1-113^. Thus, Th-T emission (Figure 2A) and Th-T kinetics (Figures 2C,E; Supplementary Figure S1A) experiments show that Stm1_N^1-113^ forms concentration-dependent amyloid conformation at 10 mM NaCl concentration, in conformity with the CD spectroscopy (Figure 1). Notably, higher concentrations (50, 100, 200, and 400 μM) exhibit a lag-phase time of about 6–12 h.

The Th-T emission (Figure 2B) and Th-T kinetics (Figures 2D,F; Supplementary Figure S1B) experiments carried out at the physiological salt concentration of 150 mM NaCl further confirm the concentration-dependent amyloidogenic characteristics of Stm1_N^1-113^ as observed in the case of 10 mM NaCl (Figures 2A,C). For instance, Th-T emission spectra show that from 50 μM Stm1_N^1-113^ concentration onwards, the fluorescence intensity increases around ~495 nm. Although the fluorescence intensity corresponding to Th-T kinetics of Stm1_N^1-113^ in the presence of 150 mM NaCl indicates amyloid fibrillation, the kinetics of fibrillation differs significantly from 10 mM NaCl at the higher concentrations (50, 100, 200, and 400 μM). The fluorescence intensities are nearly similar at these higher concentrations in the presence of 150 mM NaCl, with a steady increase in the intensities from the beginning and gradually reaching saturation after 36 h. This indicates the difference in amyloidogenic kinetics of Stm1_N^1-113^ in the presence of 10 and 150 mM NaCl. Further, the statistical analysis indicates concentration and time-dependence in amyloid formation beyond a threshold concentration of Stm1_N^1-113^ (Figures 2E,F) and time (Supplementary Figures S1A,B) in both 10 and 150 mM NaCl conditions.

### AFM images indicate the amyloid fibrillation of Stm1_N^1-113^ through rod-shaped protofibril intermediate

**Figure 3:**
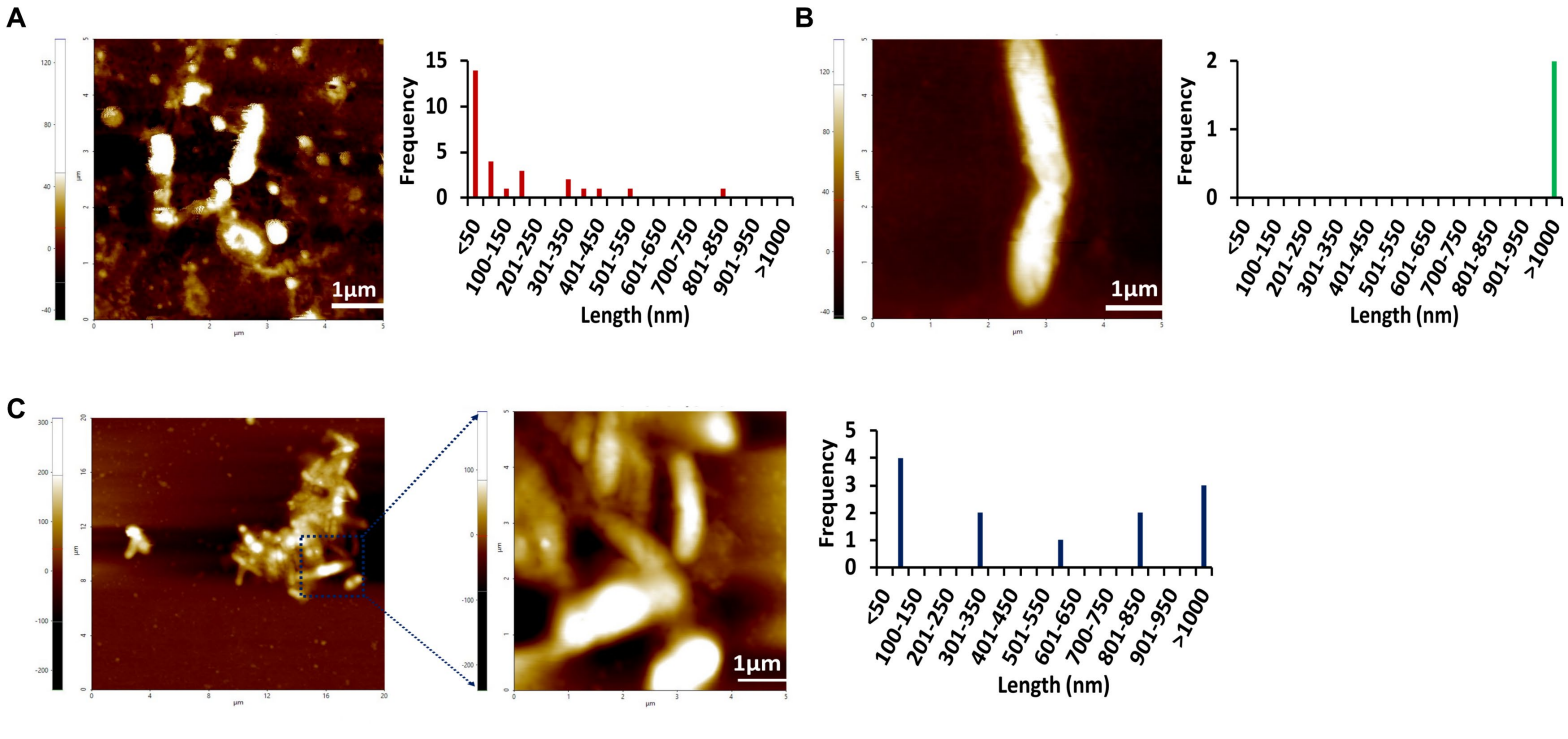
AFM images of Stm1_N^1-113^ at 400 μM concentration collected at different times **(A)** 0 h, **(B)** 24 h, and **(C)** 48 h in the presence of 10 mM NaCl indicate its amyloidogenic characteristics. The frequency and their corresponding lengths are shown alongside. The 20 μm × 20 μm scan shown in **(C)** indicates the presence of the cluster of rod-shaped protofibrils. Note that the frequency vs. height bar plot is given in Supplementary Figure S2.

**Figure 4:**
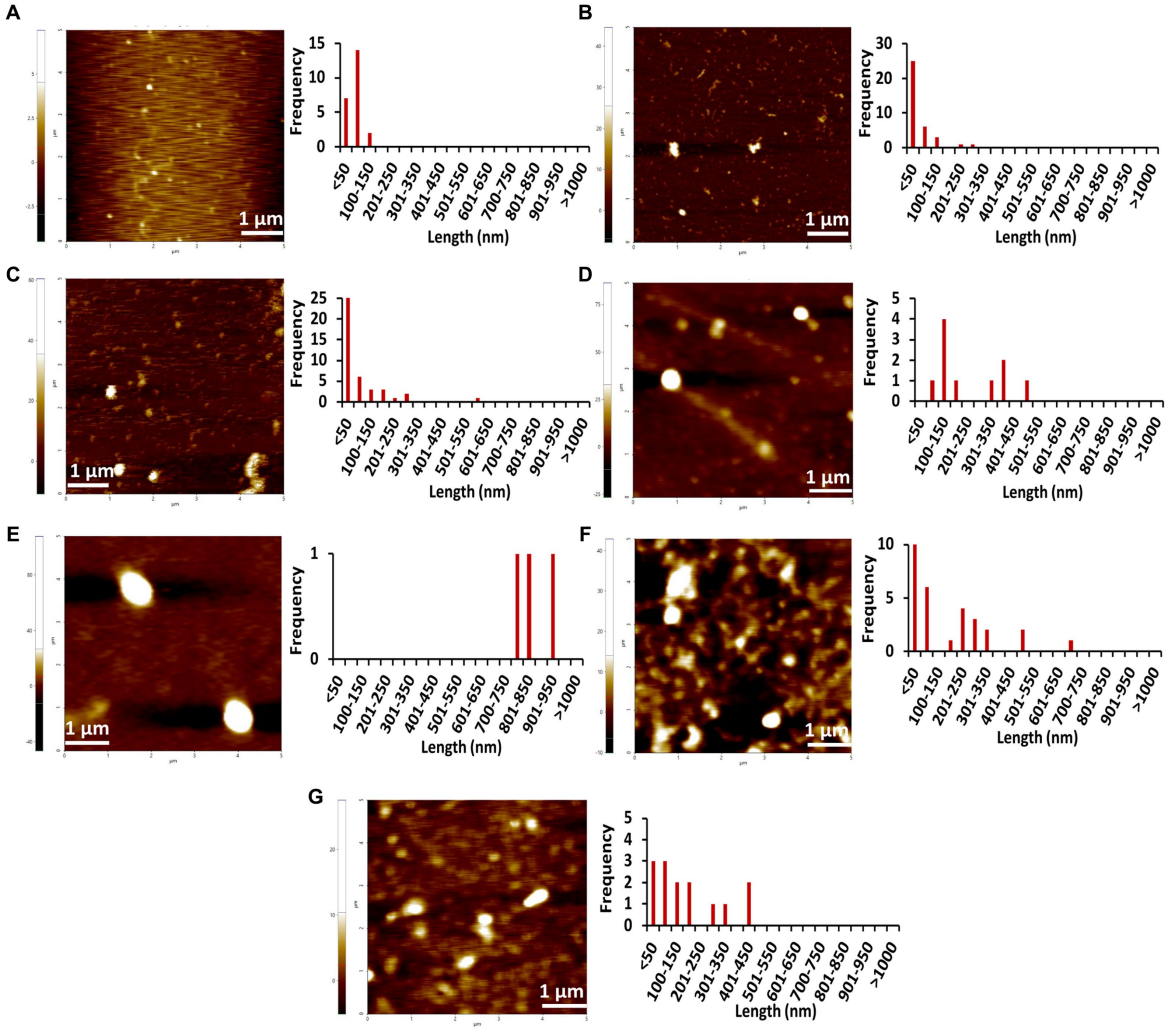
AFM images of different concentrations of Stm1_N^1-113^ collected at 0 h in the presence of a physiological concentration of 150 mM NaCl indicating the step-wise amyloid formation: **(A)** 1 μM, **(B)** 10 μM, **(C)** 20 μM, **(D)** 50 μM, **(E)** 100 μM, **(F)** 200 μM, and **(G)** 400 μM concentrations of Stm1_N^1-113^ indicate. Note that the frequency vs. height bar plot is given in Supplementary Figure S3.

**Figure 5:**
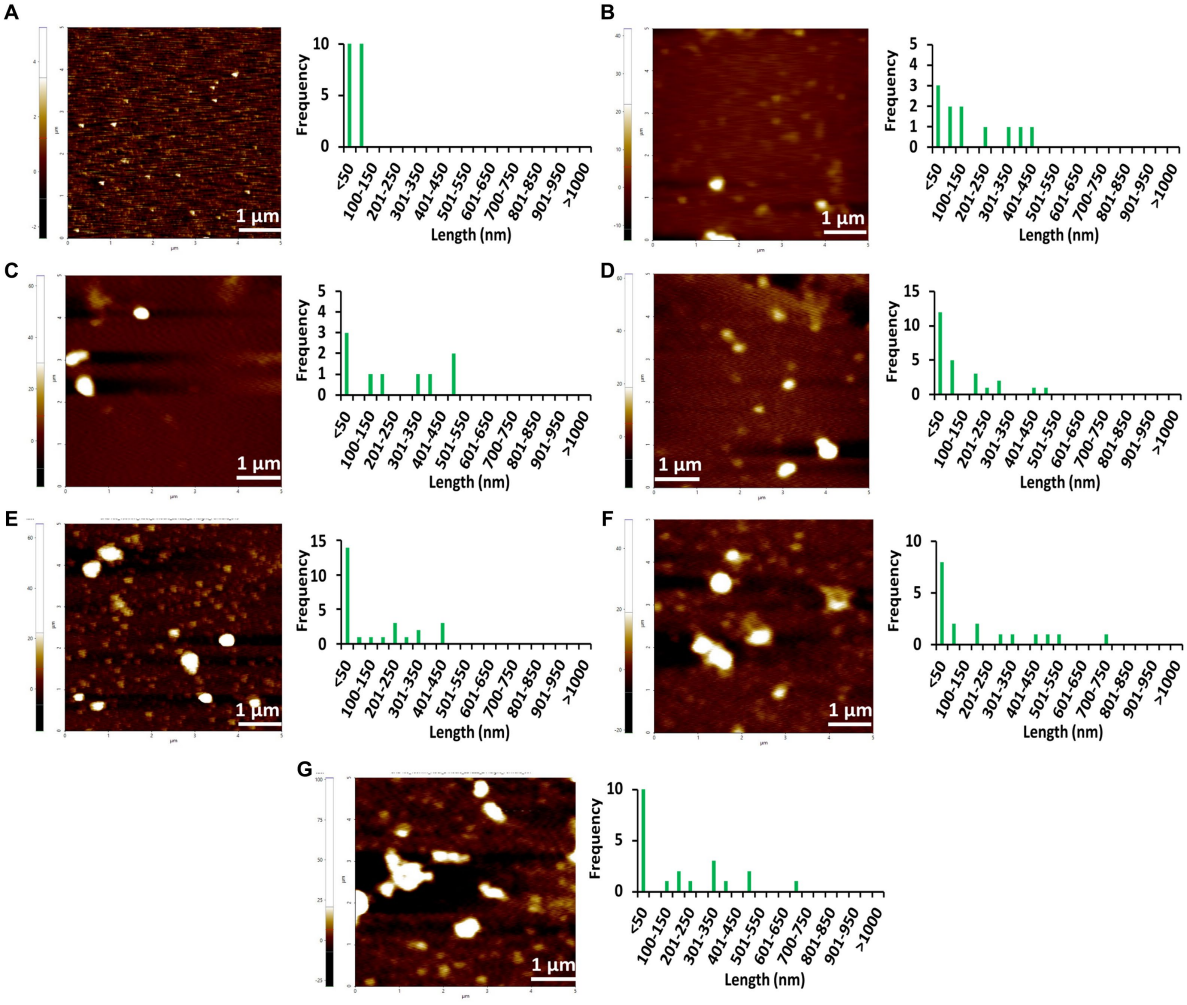
AFM images of different concentrations of Stm1_N^1-113^ collected at 24 h in the presence of a physiological concentration of 150 mM NaCl indicating the step-wise amyloid formation: **(A)** 1 μM, **(B)** 10 μM, **(C)** 20 μM, **(D)** 50 μM, **(E)** 100 μM, **(F)** 200 μM, and **(G)** 400 μM concentrations of Stm1_N^1-113^ indicate. Note that the frequency vs. height bar plot is given in Supplementary Figure S4.

**Figure 6:**
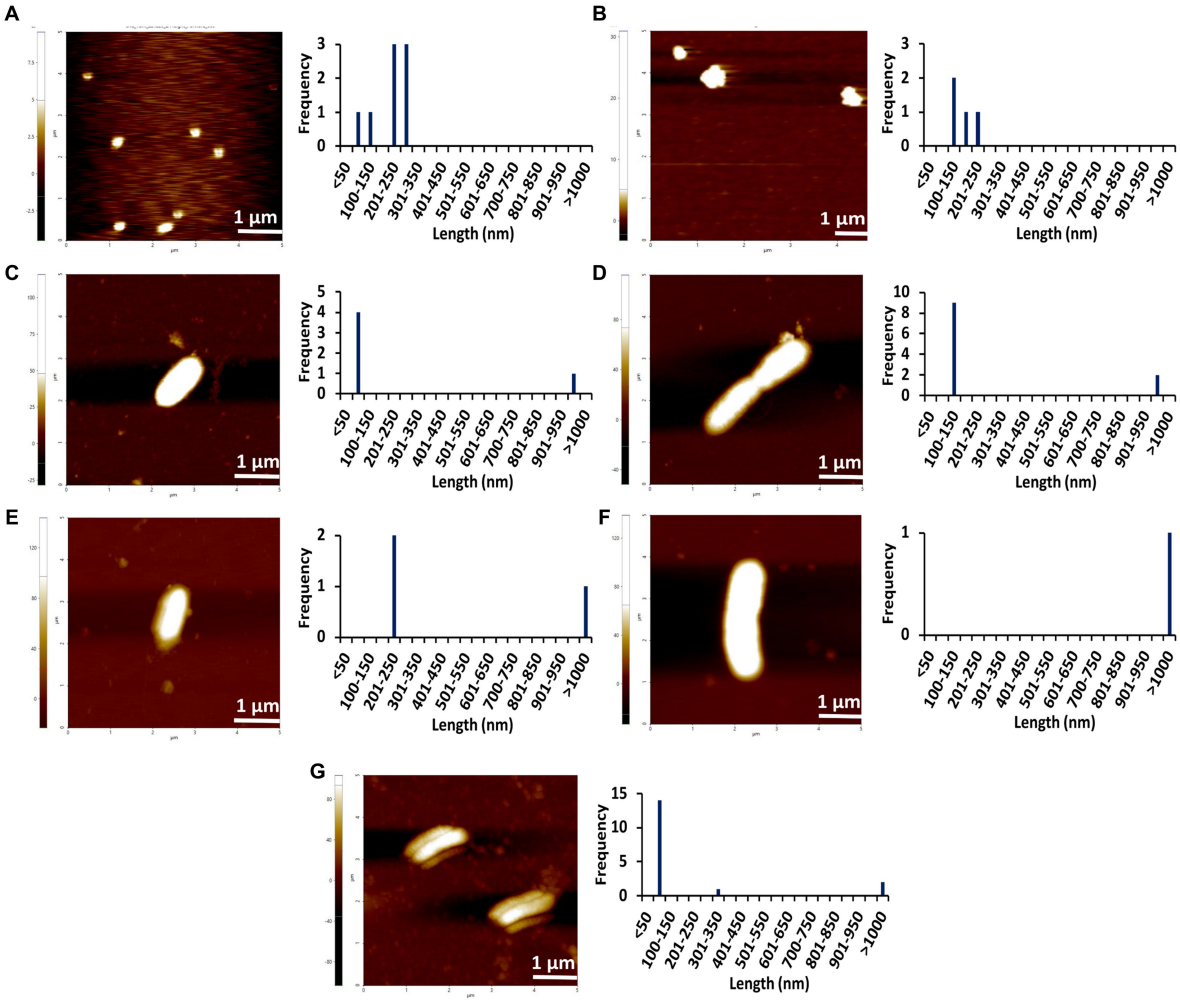
AFM images of different concentrations of Stm1_N^1-113^ collected at 48 h in the presence of a physiological concentration of 150 mM NaCl indicating the step-wise amyloid formation: **(A)** 1 μM, **(B)** 10 μM, **(C)** 20 μM, **(D)** 50 μM, **(E)** 100 μM, **(F)** 200 μM, and **(G)** 400 μM concentrations of Stm1_N^1-113^. Note that the frequency vs. height bar plot is given in Supplementary Figure S5.

To further confirm the amyloidogenic characteristics of Stm1_N^1-113^, AFM images have been collected for the 400 μM concentration of Stm1_N^1-113^ in the presence of 10 mM NaCl (Figure 3; Supplementary Figure S2) and from the lower to higher concentrations in the presence of 150 mM NaCl (Figures 4–6; Supplementary Figures S3–S5) at 0 h (Figure 4; Supplementary Figure S3), 24 h (Figure 5; Supplementary Figure S4), and 48 h (Figure 6; Supplementary Figure S5). In the presence of 10 mM NaCl (Figure 3; Supplementary Figure S2) at 0 h, AFM shows the presence of granular oligomers, spherical oligomers, and proto-fibril intermediates (Figure 3A; Supplementary Figure S2A). Although a lag phase of ~12 h in Th-T kinetics is seen in the presence of 10 mM NaCl, the AFM for 400 μM at 0 h shows the presence of granular, spherical oligomers and proto-fibrils, among which the frequency of the species (granular and spherical oligomers) having dimension lesser than 50 nm is more (Figure 3A). The lag phase seen in the Th-T kinetics could be attributed to the dominant species seen at 0 h for 400 μM Stm1_N^1-113^ concentration (Figure 2C). Such heterogeneous aggregation species, including fibrils are seen in general during the lag phase (Arosio et al., 2015). AFM image at 400 μM Stm1_N^1-113^ concentration explains the significant difference seen in CD (Figure 1), Th-T emission (Figure 2A), and Th-T kinetics (Figure 2C) spectra. For 400 μM Stm1_N^1-113^ concentration at 24 h, lengthwise merging of rod-shaped fibrils is seen with each having the dimensions of 120–150 nm height and 1.5 um length (Figure 3B; Supplementary Figure S2B). A cluster of rod-shaped fibrils are seen in the case of AFM images collected after 48 h, wherein well-defined rod-shaped fibrils each having a dimension in the range of 100–150 nm height and 1.5 um length are observed at 400 um Stm1_N^1-113^ concentration (Figure 3C; Supplementary Figure S2C). These indicate the amyloidogenic characteristics of Stm1_N^1-113^ at the 400 μM concentration.

The AFM imaging of Stm1_N^1-113^ for the concentrations of 1, 10, 20, 50, 100, 200, and 400 μM in the presence of physiological salt concentration of 150 mM NaCl at 0, 24, and 48 h has shown larger dimensions of the spherical oligomer and rod-shaped proto-fibril intermediates (Figures 4–6; Supplementary Figures S3–S5). A detailed analysis of the dimensions of different Stm1_N^1-113^ species seen at different time and concentrations indicate the time- and concentration-dependent fibrillation. During the initial phase of fibrillation (at 0 h), the concentration and fibril length relationship is inconclusive due to the presence of smaller to larger spherical oligomers for Stm1_N^1-113^ concentrations ranging from 1 to 200 μM (Figures 4A–F; Supplementary Figures S3A–F). Although multiple species are seen at 400 μM, a short rod-shaped fibril emerges only at this concentration (Figure 4G; Supplementary Figure S3G). For all the concentrations of Stm1_N^1-113^, the granular and spherical oligomers of smaller lengths are seen with a higher frequency at 0 h. However, for 400 μM Stm1_N^1-113^ concentration at 0 h, the number of species having a length greater than 50 nm (11 counts) is higher compared to the species having a length lesser than 50 nm (three counts). These may be the reasons for the absence of the lag phase at in Th-T kinetics. Similar species of smaller lengths are also seen for all the concentrations at both 24 h (Figure 5; Supplementary Figure S4) and 48 h (Figure 6; Supplementary Figure S5). However, the rod-shaped proto-fibril appears only after a threshold concentration. For instance, rod-shaped species having lengths greater than 500 nm are seen only for 200 and 400 μM concentrations at 24 h (Figures 5F,G). Such a longer proto-fibril of length greater than 500 nm starts appearing at a relatively lower concentration of 20, 50, and 100 μM at 48 h (Figures 6C–E). Nonetheless, length-wise and width-wise multimerization of the proto-fibrils is seen only at 200 μM (~3 μM length) and 400 μM (~1.5 μM length) concentrations (Figures 6F,G). These pinpoint that a threshold time and the concomitant concentration are required for the proto-fibril and fibril formation of Stm1_N^1-113^.

The AFM images of Stm1_N^1-113^ collected in the presence of 1% SDS (Sodium Dodecyl Sulfate) for 100 and 400 μM concentrations at 24 and 48 h still show the presence of Stm1_N^1-113^ fibrils (Supplementary Figure S6). However, the multimerization of the fibrils such as the ones seen for 400 μM concentration at 48 h (Figure 6G) is absent in the presence of 1% SDS (Supplementary Figure S6), although stable units of Stm1_N^1-113^ fibrils with length > 1 μm are seen in the AFM images at 48 h. In case of 100 μM concentration, ~0.8 μm long protofibrils are seen at 48 h. In any case, these images confirm the amyloidogenic characteristics of Stm1_N^1-113^ (Supplementary Figure S6).

Thus, CD, Th-T assay, and AFM results clearly indicate that Stm1_N1-113 undergoes concentration and time-dependent amyloid formation, which is pronounced at the 150 mM NaCl concentration compared to the 10 mM NaCl concentration.

## Discussion

*Saccharomyces cerevisiae* Stm1 protein is involved in several cellular events like transcription (Nelson et al., 2000), translation (Balagopal and Parker, 2011), and cell cycle (Nelson et al., 2000). It is also a well-known purine motif DNA triplex (Nelson et al., 2000) and quadruplex (Frantz and Gilbert, 1995; Van Dyke et al., 2004) binding protein. Intriguingly, the Stm1 N-terminal region (specifically, the first 80 amino acids of the N-terminus) is also found in fungal and plant proteins (Pfam ID: PF09598, a total of 641 species), wherein most of the proteins have mRNA binding domain. Further, Stm1_N^1-113^ is found to have a role in the preservation of ribosomes under nutrient deprivation environment (Van Dyke et al., 2013). Since the secondary structure of Stm1_N^1-113^ in the apo-form is unknown, the secondary structure of Stm1_N^1-113^ is investigated here by employing CD, Th-T assay, and AFM techniques.

### Amyloidogenic nature of Stm1_N^1-113^

**Figure 7:**
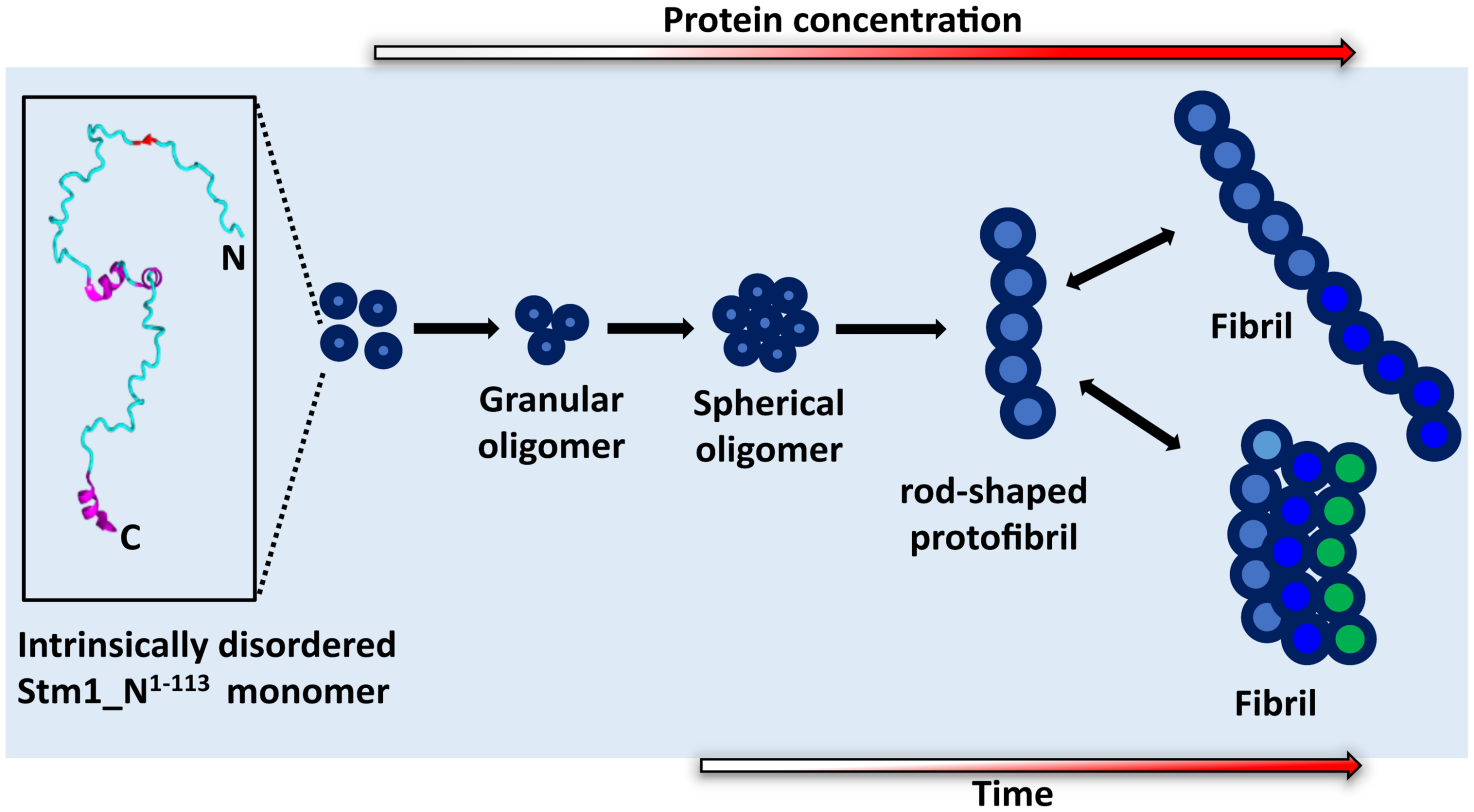
Proposed mechanism of Stm1_N^1-113^ amyloid formation. The cartoon diagram represents the intrinsically disordered Stm1_N^1-113^ present in the crystal structure of the Stm1-ribosome complex (PDB ID: 4 V88). Gradient colored arrows indicate the time and concentration dependency in the amyloid formation of Stm1_N^1-113^.

Circular dichroism spectra collected at 0 and 24 h in the presence of 10 mM NaCl indicate that Stm1_N^1-113^ exhibits concentration-dependent conformational features. For instance, it exhibits the features of intrinsically disordered conformation at the lower concentration (Figure 1A). This is in conformity with the crystal structure of the Stm1-ribosome complex, wherein the monomeric form of the Stm1 N-terminal domain possesses intrinsically disordered conformation (Figure 7). However, with the increasing concentrations of Stm1_N^1-113^, it exhibits the conformational features of β-sheet as indicated by a red shift in the negative peak (Figure 1A). Such β-sheet characteristics seen in the CD spectra, along with the enrichment of negatively charged, positively charged and hydrophobic amino acids in Stm1_N^1-113,^ provide a clue that it can take an amyloid conformation. It is noteworthy that the three short alpha helical fragments seen in Stm1_N^1-113,^ monomer encompassing the amino acid stretches 55–63, 67–73, and 101–113, may also have also taken the beta-sheet conformation. The CD spectra collected at 24 h (Figure 1B) for different Stm1_N^1-113^ concentrations eventually reflect these characteristics but with a more red shift in the negative peak and the emergence of a prominent positive peak around 195 nm at the 400 μM concentration. This suggests the time-dependent and concentration-dependent propensity to beta-sheet conformation. Th-T emission spectra collected in the presence of 10 and 150 mM NaCl to confirm the amyloidogenic characteristics show that the fluorescence intensity increases at ~495 nm above 50 μM Stm1_N^1-113^ concentrations (Figures 2A,B). This is because, Stm1_N^1-113^ may oligomerize beyond 50 μM concentrations. Such characteristics of Th-T upon binding with amyloid are well established (Biancalana and Koide, 2010; Wolfe et al., 2010; Xue et al., 2017). Th-T kinetics assay shows concentration-dependent and time-dependent amyloid formation of Stm1_N^1-113^ at 10 mM NaCl (Figure 2E; Supplementary Figure S1A) and the physiological salt concentration of 150 mM NaCl (Figure 2F; Supplementary Figure S1B).

### Amyloid fibrillation of Stm1_N^1-113^ via spherical oligomer and rod-shaped protofibril intermediates

Atomic force microscopy images collected at 0, 24, and 48 h for different concentrations of Stm1_N^1-113^ in the presence of 150 mM NaCl (Figures 4–6) illustrates a stepwise fibrillation mechanism as discussed below. Stm1_N^1-113^ granules are seen at 1 μM concentration [except at 48 h in the presence of 150 mM NaCl, wherein spherical oligomers are seen (Figure 6A)]. At the physiological salt concentration of 150 mM NaCl, the spherical oligomers and rod-shaped protofibrils appear even at the lower concentrations in a time-dependent manner (Figures 4–6; Supplementary Figures S3–S5). For instance, the proto-fibrils (~1 μm length) start appearing in the presence of 150 mM NaCl at 20 μM Stm1_N^1-113^ concentration at 48 h (Figures 6C–E). However, only spherical oligomers are seen at 0 and 24 h for the same concentration (Figures 4C–E, 5C–E). Similarly, the fibrils are seen only at the higher concentrations in a time-dependent manner: for 400 μM of Stm1_N^1-113^ at 24 h and for 200 and 400 μM at 48 h. This indicates the optimal time and concentration requirements for Stm1_N^1-113^ to exhibit amyloidogenicity. Further, a critical look at the AFM images collected at 48 h in the presence of 150 mM NaCl (Figure 6) indicates that the fibrillation can take place in two different pathways (Figure 7), *viz.*, the dimension of the fibril increases either in length-wise or width-wise manner due to the length-wise or width-wise multimerization of protofibrils, respectively. Due to the length-wise multimerization of the proto-fibrils, the length of the fibril seen at 200 uM concentration is longer (~3 μm length; Figure 6F) than the protofibrils seen at 20, 50, and 100 μM concentrations (~1 μm length) of Stm1_N^1-113^. However, the width-wise multimerization seen at 400 μM (~1.25 μm length) mimics the protofibril in terms of length, while the width is more than that of the protofibril (Figure 6G). These suggest that Stm1_N^1-113^ fibrillation takes place through the following pathway (Figure 7): formation of granules, spherical oligomers, merging of spherical oligomers to form rod shaped proto-fibrils and, length-wise or width-wise multimerization of protofibrils to form the amyloid fibrils. It is worth mentioning here that the granular inclusions of 150 mM NaCl itself (Supplementary Figure S7) may have an influence on the Stm1_N^1-113^ aggregations only at the lower concentrations such as 1 and 10 μM due to nearly similar dimensions. However, it may have a minimal influence on the multimerization of fibrils seen at the higher concentrations, as dimensions of these fibrils are much bigger (in the order of μm scale) than the salt inclusions (in the order of nm scale). Thus, AFM images reveal concentration- and time-dependency in the formation of Stm1_N^1-113^ amyloid fibrils. Further, the retention of amyloidogenic characteristics of Stm1_N^1-113^ in AFM images in the presence of 1% SDS for 100 and 400 μM concentrations at 24 and 48 h confirm its amyloid prone nature (Supplementary Figure S6).

### Enrichment of hydrophobic amino acids in Stm1_N^1-113^ may facilitate amyloid formation

A detailed inspection of the Stm1_N^1-113^ sequence composition indicates that it is enriched with positively (lysine and arginine) and negatively (aspartate and glutamate) charged amino acids. Surprisingly, the hydrophobic amino acids Ala, Val, and Gly, which are generally deficient in the intrinsically unstructured proteins (Uversky et al., 2000; Rawat and Biswas, 2012), are also significantly present Stm1_N^1-113^. While the presence of positively and negatively charged amino acids can be attributed to their intrinsically disordered nature (Uversky et al., 2000; Schlessinger et al., 2007) at the lower concentrations, the presence of hydrophobic amino acids may be the driving force for Stm1_N^1-113^ amyloid aggregation at the higher concentrations (Figures 1–7). In line with this, an earlier investigation has suggested that a high proportion of hydrophobic amino acids may make a protein excessively aggregation-prone (Sabate et al., 2015).

### Impact of Stm1_N^1-113^ structural preferences in regulating diverse biological functions

The intrinsically disordered nature of *Saccharomyces cerevisiae* Stm1_N^1-113^ may facilitate diverse biological functions of Stm1 by taking up a variety of conformations upon binding with different substrates. For instance, it takes unstructured conformation (with the presence of minor alpha helical fragments) when it complexes with *Saccharomyces cerevisiae* ribosome to preserve the ribosome under nutrition deprived conditions (Van Dyke et al., 2006, 2013; Ben-Shem et al., 2011). Indeed, *Arabidopsis thaliana* RNA-Binding Protein AtRGGA, which regulates tolerance to salt and drought stress, also has Stm1_N^1-113^ domain (Ambrosone et al., 2015). One can envisage that Stm1_N^1-113^ domain in AtRGGA may also have a similar role in preserving ribosome under stress conditions by taking an unstructured conformation.

Stm1 is also shown to play a role in apoptosis-like cell death in *Saccharomyces cerevisiae* (Ligr et al., 2001). The concentration-dependent structural preference of Stm1_N^1-113^ (intrinsically disordered to amyloid fibril formation) might be one of the factors for such apoptosis-like cell death. This is evident from the enrichment of positively charged, negatively charged and hydrophobic amino acids present in Stm1_N^1-113^, which may dictate the random coil to amyloid fibril formation at different concentrations, thus, facilitating the diverse biological functions of Stm1. The amyloid fibril conformation seen in the AFM images at the higher concentrations of Stm1_N^1-113^ in the presence of 150 mM NaCl (Figures 4–6) indicates the perpetuating nature of intrinsically unstructured Stm1_N^1-113^ monomer (Figure 7). Indeed, an earlier bioinformatics analysis has suggested that the intrinsically disorder regions have a sufficient balance between amino acids that provide intrinsically disordered characteristics as well as facilitate the amyloid formation (Sabate et al., 2015). More specifically, asparagine/glutamine are found to dominantly occur in such intrinsically disorder regions to facilitate amyloid conformation (Michelitsch and Weissman, 2000; Uptain and Lindquist, 2002; Alberti et al., 2009). Since several proteins (such as aβ42, alpha-synuclein, etc.) whose amyloid-forming ability of N- or C-termini influences the full-length protein, thus, the associated disease (Mirza et al., 2014; Chen et al., 2017; Li et al., 2018), one can also envisage similar effect in Stm1 full-length protein. Further, the amyloid fibrillation of Stm1_N^1-113^ seen at the higher concentration suggests that in response to environmental stress and/or cellular signaling, the cell may overexpress Stm1 and induce cytotoxicity due to the amyloid fibril formation of Stm1_N^1-113^. Under stress-induced conditions, overexpression of Stm1 protein has led to apoptosis-like cell death in *Saccharomyces cerevisiae* (Ligr et al., 2001). Further, such cytotoxicity mediated by amyloid fibril formation is a well-established phenomenon (Demuro and Parker, 2013; Marshall et al., 2014; Pezza et al., 2014; Rencus-Lazar et al., 2019). It is noteworthy that a similar mechanism has been identified in yeast translation termination factor Sup35, wherein the prion domain of intrinsically disordered N-terminal region protects Sup35 by forming a visible gel-like condensate upon pH stress (Franzmann et al., 2018). Nevertheless, Stm1_N^1-113^ forms well-defined amyloid fibril (Figures 3–6) which might be irreversible (unlike Sup35 condensate), thus may lead to cell death. Such amyloid mediated apoptosis has been getting attention in recent years (Han et al., 2017; Takada et al., 2020). It is noteworthy that unlike Orb2 of *drosophila melanogaster*, wherein the mRNA binding domain is different from the amyloid forming domain, the mRNA binding N-terminal domain of Stm1 itself exhibits amyloidogenic character (Hervas et al., 2020).

In summary, Stm1_N^1-113^ which is a purine motif DNA triplex and G-quadruplex binding domain of *Saccharomyces cerevisiae* Stm1 protein, is shown here to form concentration and time-dependent amyloid morphology by employing CD, Th-T assay, and AFM experiments.

## Materials and methods

### Sub-cloning of Stm1_N^1-113^ into pET-21a expression vector

To sub-clone *stm1* gene (YLR150W) of *Saccharomyces cerevisiae* (S288c) in pET-21A vector, ampicillin resistant pBG1805 cloning vector which has the *stm1* gene (Supplementary Figure S8) was purchased from Thermofisher Scientific Inc. The region of *stm1* gene, which encodes for Stm1_N^1-113^ was amplified using the appropriate forward (5′GGAATTCCATATGTCCAACCCATTTGATTTGTTAGG3′) and reverse (5′ ACGCGTCGACTTGTCATCACCCCAACCTTGGTTAAC3′) primers that have Nde1 and Sal1 restriction sites, respectively. Subsequently, the amplified PCR product was subjected to double digestion and was ligated into an ampicillin-resistant pDZ1 expression vector (a modified form of pET-21A vector that has the T7 promoter; Rathinavelan et al., 2011, 2014). Finally, the construct was verified with the help of DNA sequencing (Supplementary Figure S8). The resultant construct consisted of regions that can encode for Stm1_N^1-113^ tagged with His_6_ and GB1 tags in the following sequential order: N-terminal His_6_-tag, GB1 solubility tag, tobacco etch virus (TEV) protease cleavage site and Stm1_N^1-113^. Including the termination codon, the sequence inserted into pET-21a vector was 342 nucleotides in length.

### Protein expression and purification

The abovementioned pDZ1 expression vector, which can code for Stm1_N^1-113^ was transferred into *E. coli* BL21 (DE3; Bioline) cells. To overexpress the protein, the pre-inoculum cells (10 mL) were grown overnight, from which 1 ml was then transferred into 1 L of LB broth (which contains 100 mg of ampicillin). The culture was subsequently grown in an orbital shaker incubator at 37°C and 210 rpm until the optical density (OD) reached the value of 0.6. At 0.6 OD, the culture was induced with 1 mM isopropyl-thio-D-galactopyranoside (IPTG) and then incubated at 18°C overnight to attain the maximum OD. The cells were subsequently harvested and sonicated in the presence of binding buffer (20 mM Tris–HCl, 500 mM NaCl, and 5 mM imidazole, pH–8.0) with the addition of phenylmethylsulfonyl fluoride (PMSF) to prevent the action of proteases. The purification of Stm1_N^1-113^ was carried out in a two-step process with the help of Ni^2+^-nitrilotriacetate (Ni^2+^-NTA) affinity column chromatography as described elsewhere (Wang et al., 2007; Rathinavelan et al., 2011; Kolimi et al., 2017; Ajjugal et al., 2021; Ajjugal and Rathinavelan, 2021). Initially, Stm1_N^1-113^ attached with His_6_ and GB1 tags, was purified and was eluted in the elution buffer (20 mM Tris–HCl, 500 mM NaCl, and 200 mM imidazole, pH–8.0). The purified Stm1_N^1-113^ (which was attached with His_6_ and GB1 tags) was subjected to overnight TEV protease digestion to cleave the His_6_ and GB1 tags from Stm1_N^1-113^. During the second round of purification, the cleaved Stm1_N^1-113^ was collected in the binding buffer and the His_6_ and GB1 tags were collected in the elution buffer (Supplementary Figure S9). Finally, the protein was dialyzed in the phosphate buffer (10 mM sodium phosphate, 10 mM, or 150 mM NaCl, at pH 7.4). After the dialysis, Stm1_N^1-113^ was concentrated with the help of an Amicon protein concentrator (molecular weight cut-off of 3 kDa). The concentration of protein was measured at 280 nm with the help of a UV absorption spectrophotometer. The extinction coefficient value of [5,500 (mg/mL)^−1^ cm^−1^] was used to measure the concentration.

### Circular dichroism spectroscopy

Circular dichroism spectroscopy was employed to study the concentration-dependent secondary structural preference of Stm1_N^1-113^ by considering 20, 50, 100, 200, and 400 μM concentrations of Stm1_N^1-113^. The CD spectra corresponding to the various concentrations of Stm1_N^1-113^ were collected in a 0.1 mm pathlength cuvette. For each concentration, the CD spectra were collected in triplicates at 25°C in the wavelength range of 190–260 nm with a scan rate of 50 nm/min using JASCO J-1500 spectropolarimeter. Finally, the triplicate average of the CD spectra was used for the analyses. All the spectra were processed with JASCO Spectra Manager software. 10 mM sodium phosphate and 10 mM NaCl buffer (pH 7.4) were used for the baseline correction.

### Thioflavin-T assay

Thioflavin-T fluorescent dye was employed to study the concentration-dependent amyloidogenic character of Stm1_N^1-113^ by considering 1, 10, 20, 50, 100, 200, and 400 μM concentrations of Stm1_N^1-113^ in 10 mM sodium phosphate in the presence of 10 or 150 mM NaCl. The samples were loaded in an Eppendorf 96 well black Microplate with a clear film bottom, and the excitation and emission spectra were collected using EnSpire multimode plate reader (Perkin Elmer). The Th-T emission spectra were collected at 0 h by keeping the concentration of Th-T constant (20 μM) and by varying the concentration of Stm1_N^1-113^. It is well-known that the binding of Th-T to the amyloid conformation changes the excitation and emission wavelengths of Th-T to ~442 and ~485 nm, respectively (Biancalana and Koide, 2010; Wolfe et al., 2010). Thus, the emission spectra were scanned in the wavelength range of 465 to 565 nm at a bandwidth of 2 nm by keeping the excitation wavelength at 442 nm. All the experiments were carried out in the phosphate buffer (10 mM sodium phosphate with 10 or 150 mM NaCl, pH 7.4) and Th-T (20 μM) dissolved in this buffer was used as a blank. For each concentration, the spectra were collected in triplicates at 25°C, and the triplicate average of the spectra was used for further analyses.

Thioflavin-T kinetics assay was also carried out to understand the amyloid fibrillation kinetics of the above mentioned Stm1_N^1-113^ concentrations (1, 10, 20, 50, 100, 200, and 400 μM) in the presence of 10 and 150 mM NaCl. As before, Th-T emission spectra were collected in triplicates by titrating with 20 μM Th-T. The reading was collected until 72 h, and finally, the time vs. fluorescence intensity plot was derived to confirm the amyloid fibril formation. Statistical significance analysis with one-way ANOVA using Tukey *post-hoc* test was independently carried out for concentration and time-dependency using Microsoft excel and GraphPad Prism 10.0 (GraphPad, 2023). The estimation of standard error of mean (SEM) was carried out using Microsoft excel.

### Atomic force microscopy

The tapping mode atomic force microscopy (AFM) images were collected using Park Systems NX10. Collected data was analyzed with the help of XEI software. In addition, Gwyddion software was used for the quantification of (i) amyloid morphologies captured in the AFM and (ii) their dimensions (Nečas and Klapetek, 2012). AFM images were captured at 400 μM Stm1_N^1-113^ concentrations in the presence of 10 mM NaCl and 1, 10, 20, 50, 100, 200, and 400 μM concentrations in 150 mM NaCl using the commercially available mica sheet as a sample holder. Additionally, AFM images were collected for 100 and 400 μM concentrations of Stm1_N^1-113^ in the presence of 1% SDS. Prior to this the protein was incubated with SDS for 30 min. Using the following protocol, the protein sample was plated on the mica sheet. Initially, the freshly cut mica sheet was dipped in APTES [(3-Aminopropyl)triethoxysilane] solution (AP-mica) and washed as described elsewhere (Shlyakhtenko et al., 2013). The AP-mica sheet was dried in a vacuum desiccator for 2–3 h. Followed by this step, the AP-mica sheets were suspended for 30 min in 1 mL protein solution in a 1.5 mL microcentrifuge tube. The same protocol was followed for dipping the AP-mica sheets into protein-SDS solution. Subsequently, the AP-mica sheet was washed with 3 mL of double deionized water as described elsewhere (Shlyakhtenko et al., 2013), dipped into 1 mL water followed by 2 mL water to thoroughly remove the excess NaCl and dried in a vacuum desiccator for 2–3 h. This washing protocol was standardized based on the control AFM experiments carried out only for 10 and 150 mM NaCl (*viz.*, without the protein but with the phosphate buffer) which showed the significant removal of salt from the AP-mica sheet (Supplementary Figure S7). Finally, the concentration and time-dependent (0, 24, and 48 h) AFM images of Stm1_N^1-113^ were captured using the dried AP-mica sheet surface.

## Data availability statement

The original contributions presented in the study are included in the article/Supplementary material. Further inquiries can be directed to the corresponding author.

## Author contributions

TR conceptualized the project. VS executed the CD, Th-T assay, and AFM data collection. PPU optimized AFM protocol and collected time-dependent AFM data. UD carried out statistical analysis and AFM data analysis. SS helped in AFM data collection. VS, PPU, and TR wrote the manuscript. All authors contributed to the article and approved the submitted version.
