# Supplementary material for: Concentration and time-dependent amyloidogenic characteristics of intrinsically disordered N-terminal region of Saccharomyces cerevisiae Stm1
Source: Front Microbiol. 2023 Oct 19;14:1206945. doi: 10.3389/fmicb.2023.1206945 (PMC10620681; doi:10.3389/fmicb.2023.1206945)
Supplement: Supplementary file 1 [file Data_Sheet_1.docx]

**Concentration and time-dependent amyloidogenic characteristics of intrinsically disordered N-terminal region of *Saccharomyces cerevisiae* Stm1**

**Running title:** **Fibrillation of *S. cerevisiae* Stm1 N-terminal region**

Venkata Subbaiah S P‡, Patil Pranita Uttamrao‡, Uttam Das#, Sruthi Sundaresan#, and

Thenmalarchelvi Rathinavelan*

Department of Biotechnology, Indian Institute of Technology Hyderabad,

Kandi, Telangana state-502285, India

^‡^These authors contribute equally

# These authors contribute equally

^*^For correspondence: tr@bt.iith.ac.in

**Keywords:** *Saccharomyces cerevisiae*, Stm1, intrinsically disordered protein, amyloid fibril, apoptosis-like cell death, triplex binding protein, quadruplex binding protein

**The file contains supplementary Figures S1 to S9**

| **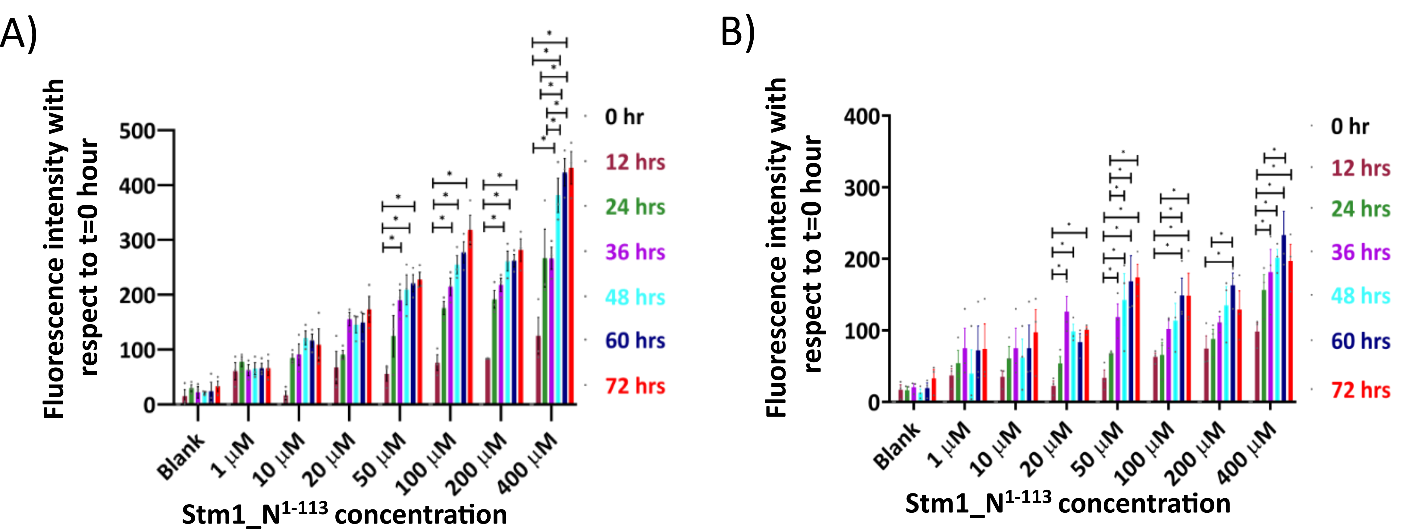** |
| --- |
| **Figure S1.** Fluorescence intensity bar diagram illustrating the time effect on Stm1_N^1-113^ aggregation in the presence of 10 mM (E) and 150 mM NaCl (F) at different protein concentrations (1 µM, 10 µM, 20 µM, 50 µM, 100 µM, 200 µM, and 400 µM) in a time interval of 12 hours during 0 to 72 hrs. Statistical significance analyzed with one-way ANOVA using the Tukey *post-hoc* test is indicated (p ≤ 0.05) along with the standard error of the mean (SEM). Note that the corresponding data points (sample size=3) are indicated in filled circles. |

| **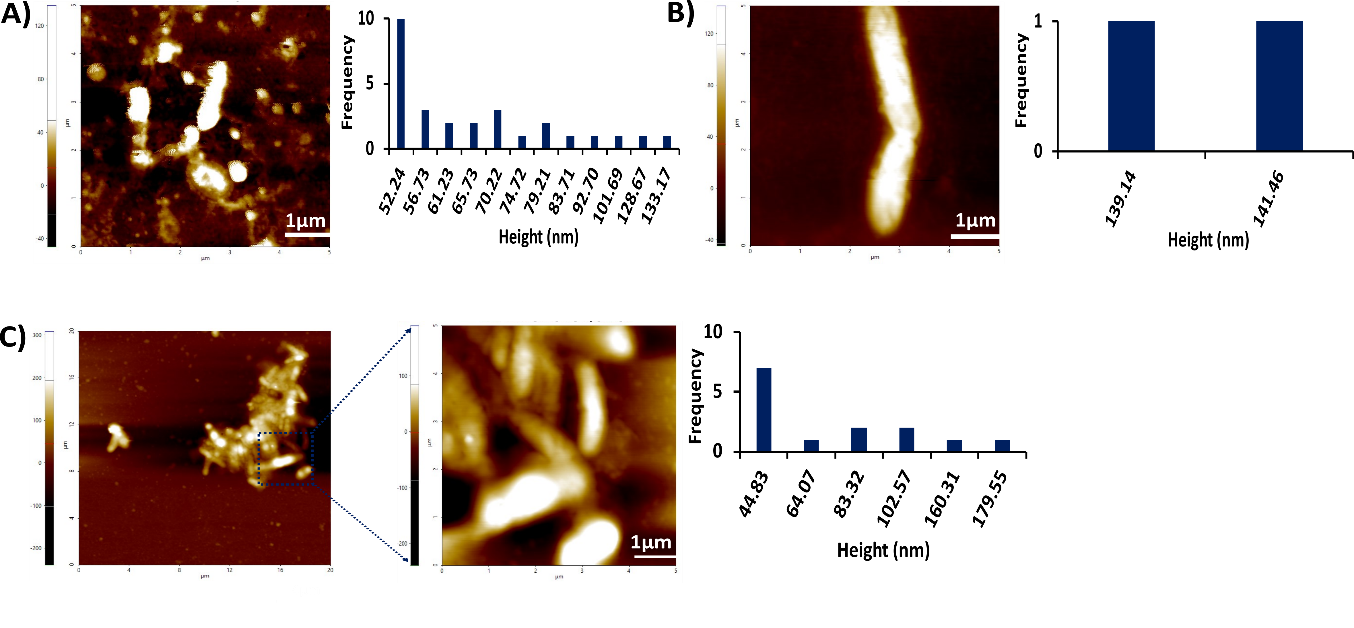** |
| --- |
| **Figure S2.** **AFM images corresponding to Figure 3.** AFM images corresponding to 400 µM Stm1_N^1-113^ concentration collected at A) 0 h, B) 24 h, and C) 48 h in the presence of 10 mM NaCl. The height (X-axis) vs frequency (Y-axis) plot is given alongside each AFM image. |

| 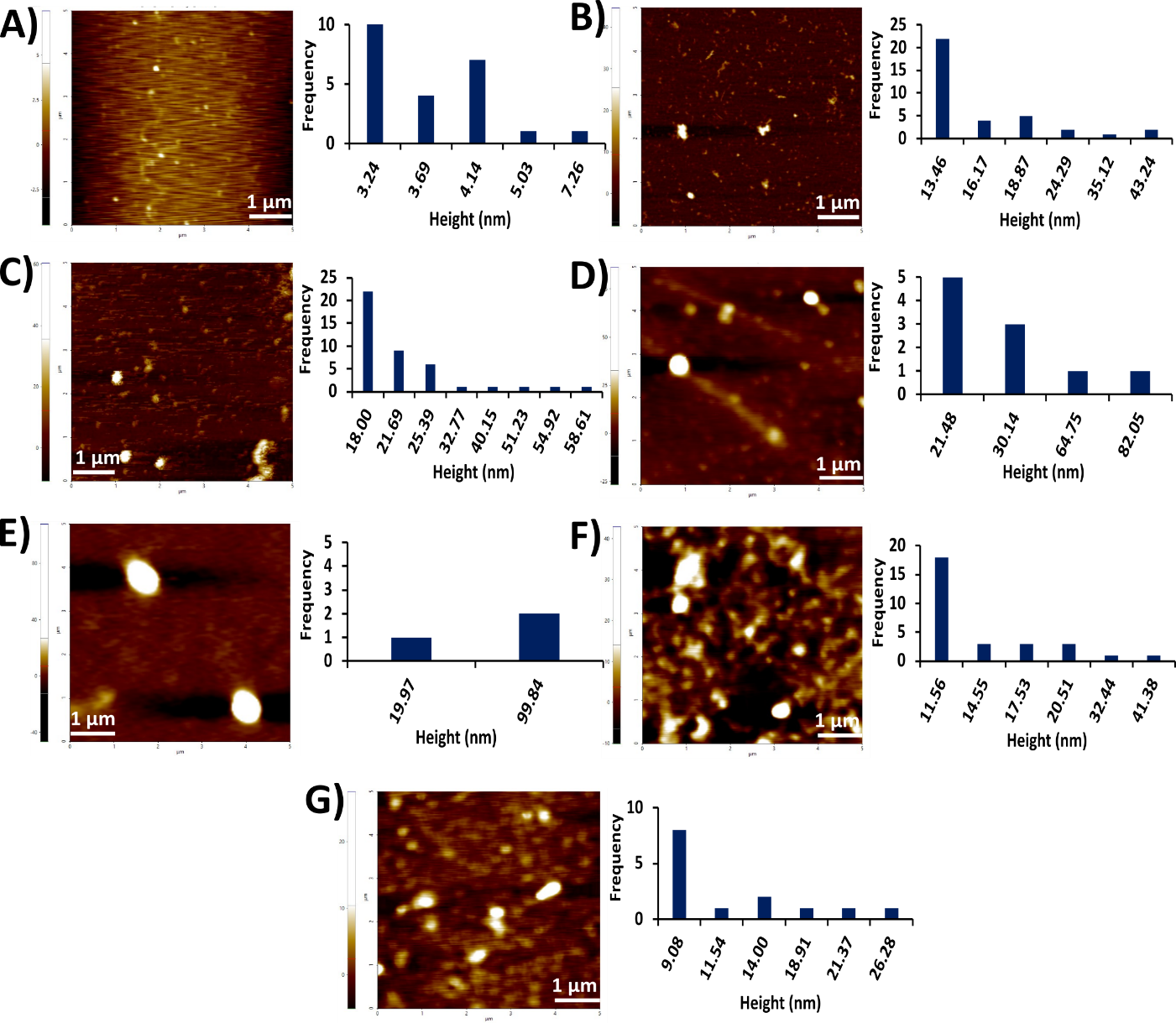 |
| --- |
| **Figure S3.** **AFM images corresponding to Figure 4.** AFM images corresponding to different Stm1_N^1-113^ concentrations collected at 0 h in the presence of 150 mM NaCl: A) 1 µM B) 10 µM C) 20 µM D) 50 µM E) 100 µM F) 200 µM and G) 400 µM. The height (X-axis) vs frequency (Y-axis) plot is given alongside each AFM image. |

| 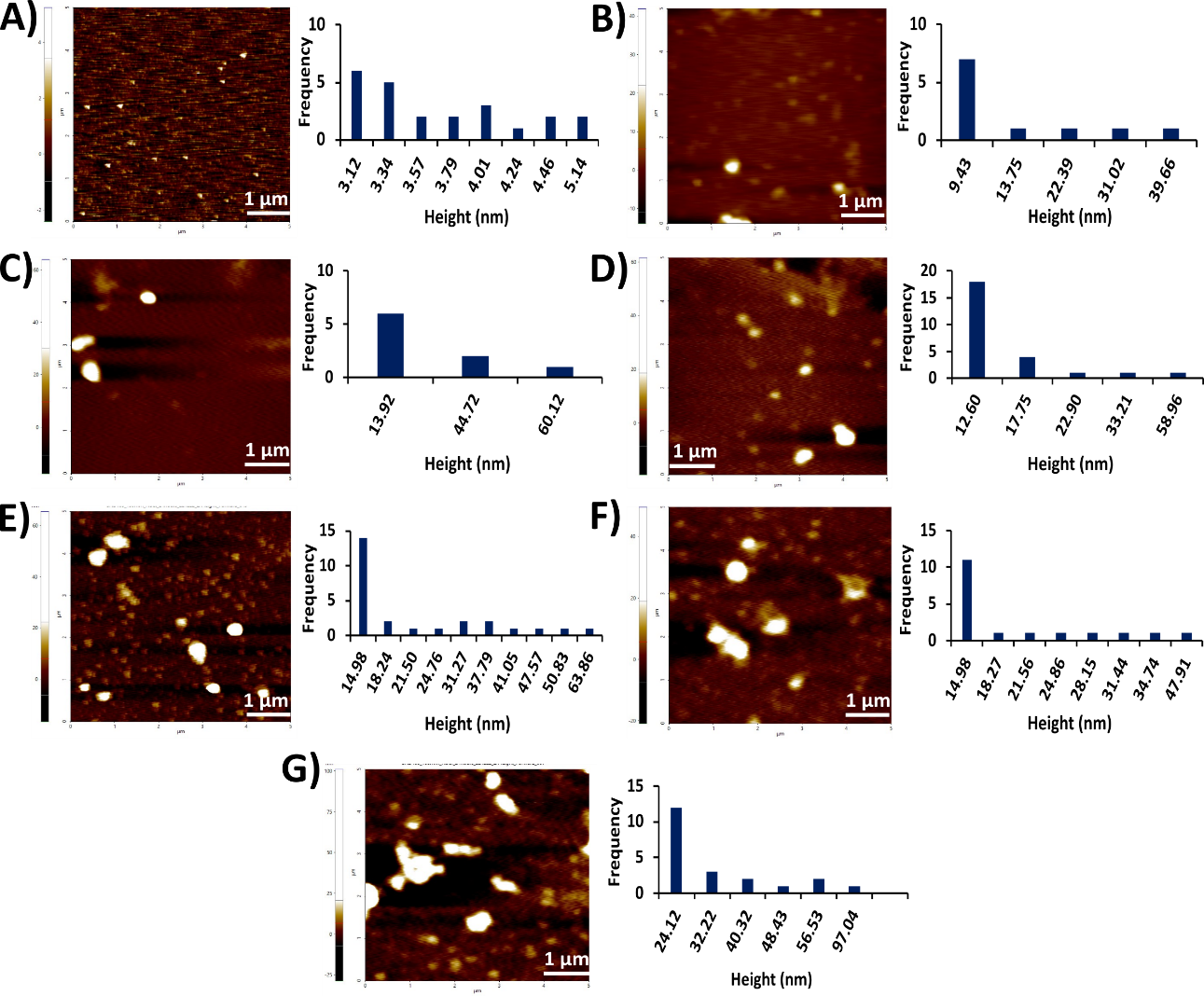 |
| --- |
| **Figure S4.** **Extended AFM images corresponding to Figure 5.** AFM images corresponding to different Stm1_N^1-113^ concentrations collected at 24 h in the presence of 150 mM NaCl: A) 1 µM B) 10 µM C) 20 µM D) 50 µM E) 100 µM F) 200 µM and G) 400 µM. The height (X-axis) vs frequency (Y-axis) plot is given alongside each AFM image. |

| 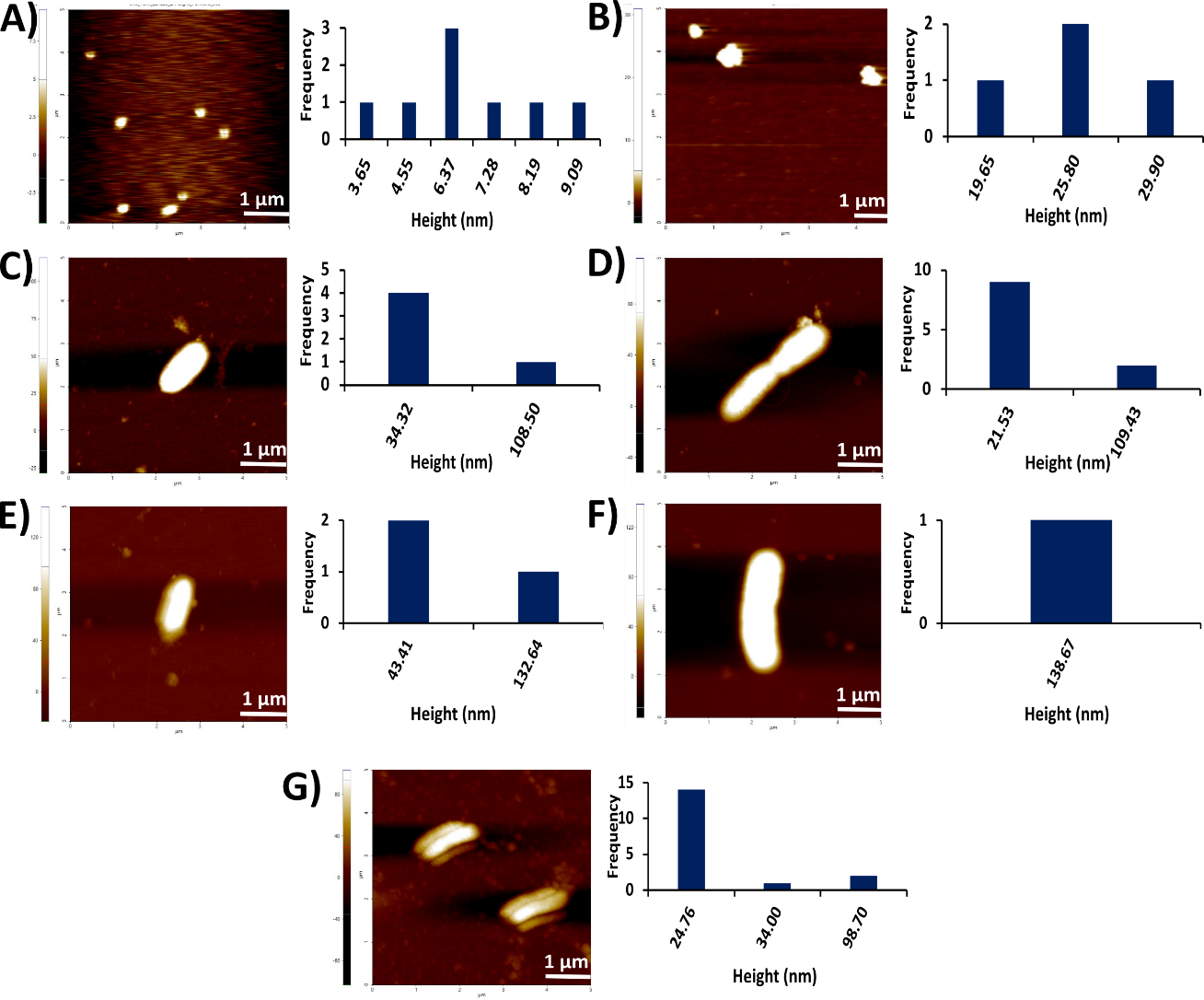 |
| --- |
| **Figure S5.** **Extended AFM images corresponding to Figure 6.** AFM images corresponding to different Stm1_N^1-113^ concentrations collected at 48 h in the presence of 150 mM NaCl: A) 1 µM B) 10 µM C) 20 µM D) 50 µM E) 100 µM F) 200 µM and G) 400 µM. The height (X-axis) vs frequency (Y-axis) plot is given alongside each AFM image. |

| 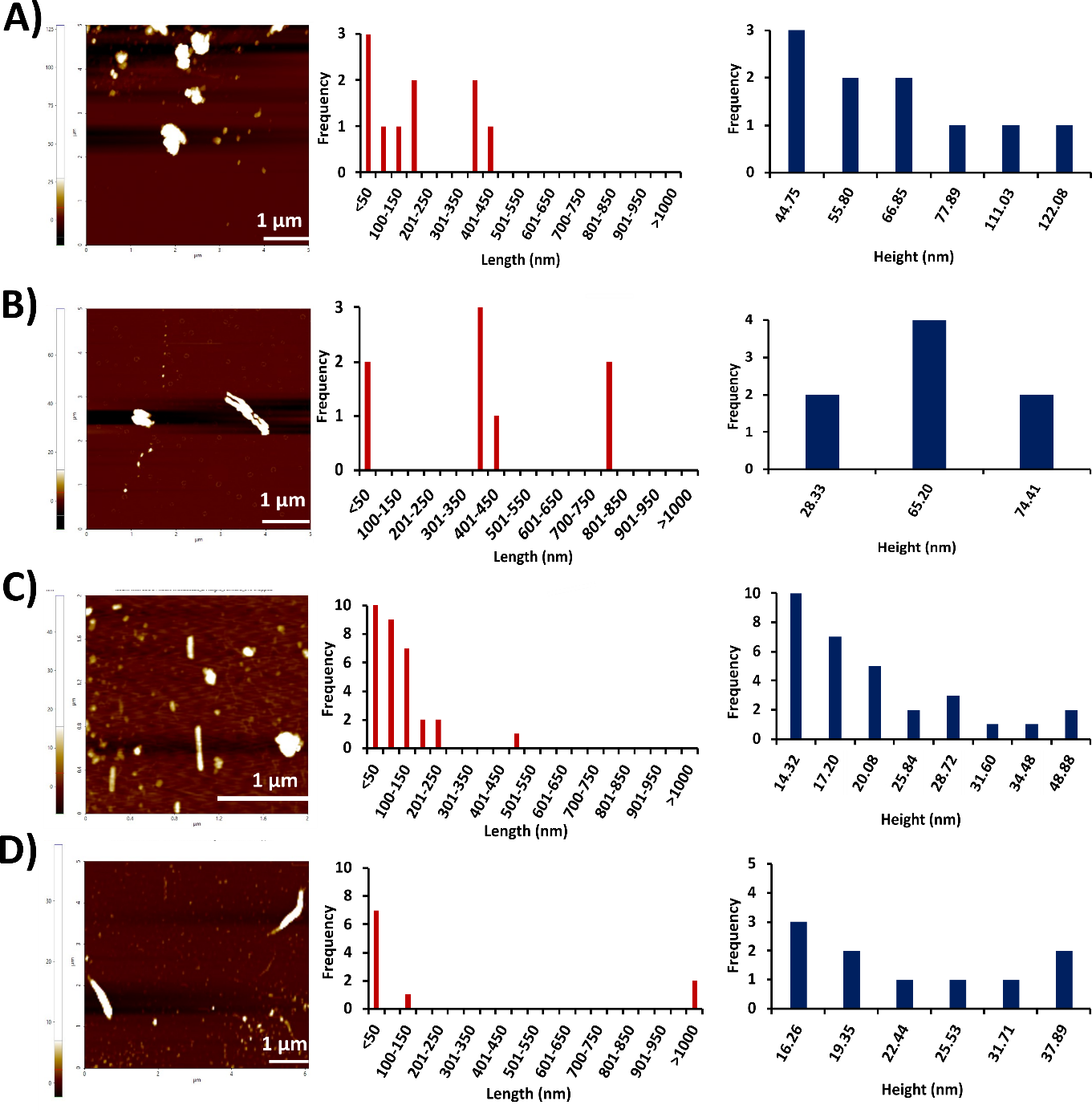 |
| --- |
| **Figure S6. AFM images corresponding to different concentrations of Stm1_N^1-113^ collected in the presence of 150 mM NaCl and 1% SDS:** A) 100 µM at 24 h, B) 100 µM at 48 h, C) 400 µM at 24 h and D) 400 µM 48 h. The length (X-axis) vs. frequency (Y-axis) and height (X-axis) vs. frequency (Y-axis) plots are given alongside each AFM image. The presence of rod-shaped amyloid morphology is seen for both concentrations at 48 h (B and D). |

| 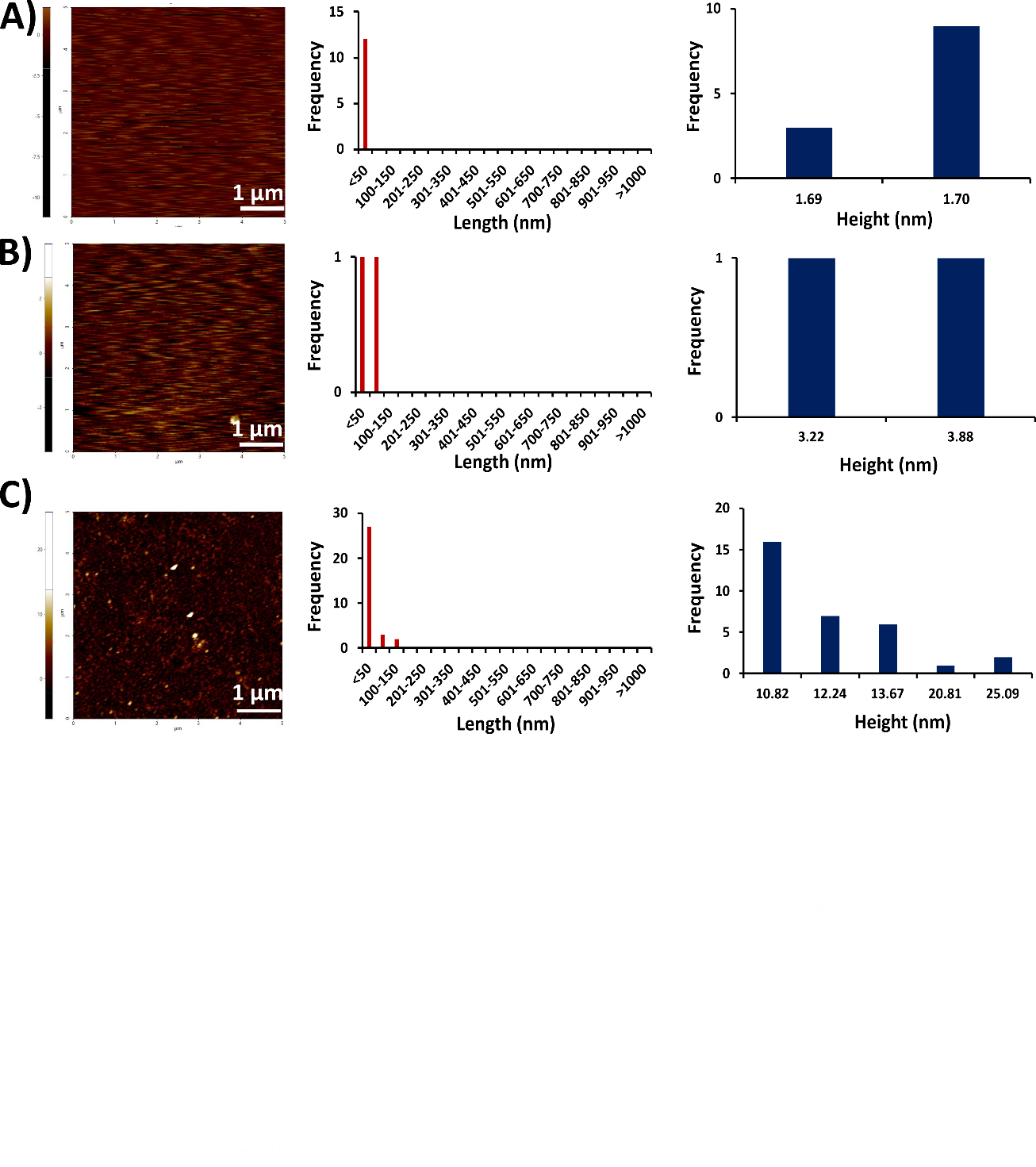 |
| --- |
| **Figure S7. AFM images were collected for 10 mM NaCl and 150 mM NaCl (in the presence of phosphate buffer, pH=7.4) alone (*viz*., without the protein):** A) Empty mica sheet (reference), B) 10 mM NaCl on APTES-coated mica sheet with 3 ml washing followed by dipping in 1 ml double deionized water and C)150 mM NaCl on APTES coated mica sheet with 3 ml washing followed by dipping in 1 ml water and again by washing with 2ml double deionized water. The length (X-axis) vs. frequency (Y-axis) and height (X-axis) vs. frequency (Y-axis) plots are given alongside each AFM image. Note that 3 ml of washing followed by dipping in 1 ml of water and again 2 ml of washing is used for the AFM image collection of Stm1_N^1-113^. |

| 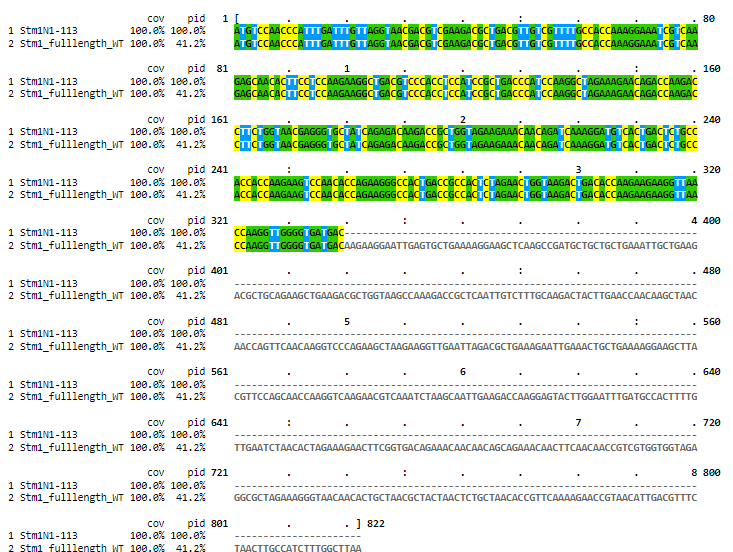 |
| --- |
| **Figure S8. Pairwise alignment of Stm1 full-length wild-type sequence (Uniprot ID:** **P39015) with the sequencing result of sub-cloned Stm1_N^1-113^.** |

| 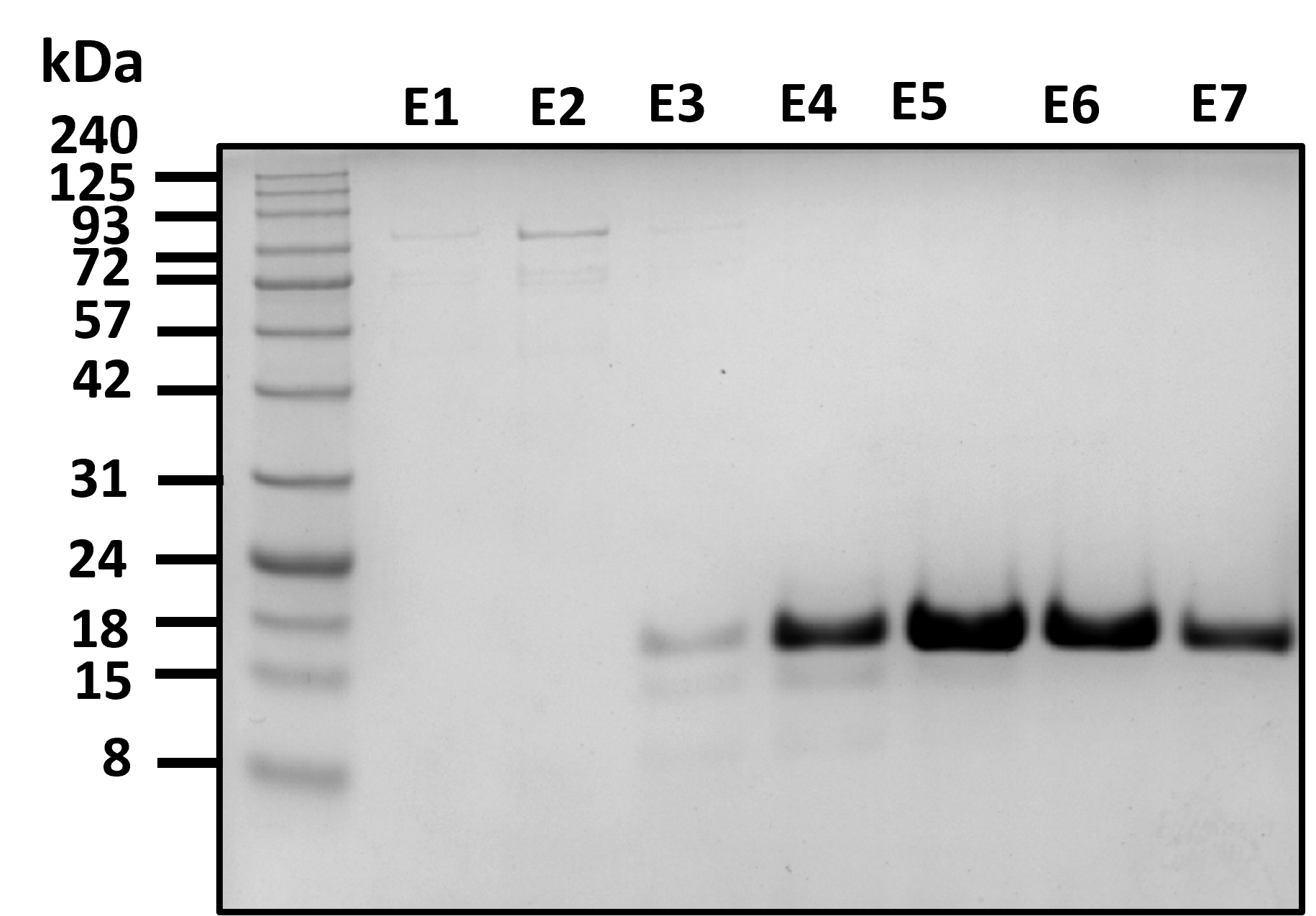 |
| --- |
| **Figure S9. Different elusions of Stm1_N^1-113^. Lane number 2 to 7:** Elusions (E1-E7; E1(2 ml), E2 (2 ml), E3 (5 ml), E4 (10 ml), E5 (10 ml), E6 (10 ml) and E7 (10 ml)) collected after the second round of purification (see Materials and Methods for the details). Pre-stained protein marker from Himedia used as a reference is shown in Lane 1. |
